# A non-bioartificial liver support system combined with transplantation in HBV-related acute-on-chronic liver failure

**DOI:** 10.1038/s41598-021-82719-x

**Published:** 2021-02-03

**Authors:** Peng Li, Xi Liang, Shan Xu, Ye Xiong, Jianrong Huang

**Affiliations:** 1 State Key Laboratory for Diagnosis and Treatment of Infectious Diseases, National Clinical Research Center for Infectious Diseases, Collaborative Innovation Center for Diagnosis and Treatment of Infectious Diseases, The First Affiliated Hospital, Zhejiang University School of Medicine, 79 Qingchun Rd, Hangzhou, 310003 China; 2 Precision Medicine Center, Taizhou Central Hospital (Taizhou University Hospital), Taizhou, 318000 China

**Keywords:** Outcomes research, Infectious diseases, Hepatitis

## Abstract

We aim to determine the impact of an artificial liver support system (ALSS) treatment before liver transplantation (LT), and identify the prognostic factors and evaluate the predictive values of the current commonly used ACLF prognostic models for short-term prognosis after LT. Data from 166 patients who underwent LT with acute-on-chronic liver failure (ACLF) were retrospectively collected from January 2011 to December 2018 from the First Affiliated Hospital of Zhejiang University School of Medicine. Patients were divided into two groups depending on whether they received ALSS treatment pre-LT. In the observation group, liver function tests and prognostic scores were significantly lower after ALSS treatment, and the waiting time for a donor liver was significantly longer than that of the control group. Both intraoperative blood loss and period of postoperative ICU care were significantly lower; however, there were no significant differences between groups in terms of total postoperative hospital stays. Postoperative 4-week and 12-week survival rates in the observation group were significantly higher than those of the control group. Similar trends were also observed at 48 and 96 weeks, however, without significant difference. Multivariate Cox regression analysis of the risk factors related to prognosis showed that preoperative ALSS treatment, neutrophil–lymphocyte ratio, and intraoperative blood loss were independent predicting factors for 4-week survival rate after transplantation. ALSS treatment combined with LT in patients with HBV-related ACLF improved short-term survival. ALSS treatment pre-LT is an independent protective factor affecting the 4-week survival rate after LT.

## Introduction

Hepatitis B virus-related acute-on-chronic liver failure (HBV-ACLF) is a severe stage of hepatitis B infection. The disease progresses rapidly and carries a mortality rate of more than 50% if standardized treatment is not received in a timely fashion^1–3^. Liver transplantation (LT) is an effective treatment of liver failure, and it has a significant effect in some large transplant centers^4,5^. The 1-year survival rate after LT is more than 80–90%. However, a critical limitation is the lack of donor livers^6,7^.

In recent decades, artificial liver support systems (ALSS) has been demonstrated to be safe and well tolerated as a bridging therapy prior to LT^8–11^. However, it remains unclear as to whether ALSS treatment pre-LT has an impact on survival rates. Therefore, the purpose of this study was to determine the impact of ALSS treatment on the surgery and outcomes after LT, and to identify prognostic factors and predictive values of the current commonly used ACLF prognostic models for short-term prognosis after LT.

## Patients and methods

### Patients

This was a retrospective study. All data were derived from patients who underwent LT with HBV-ACLF from January 2011 to December 2018 at the First Affiliated Hospital of Zhejiang University School of Medicine. HBV-ACLF was diagnosed based on APASL criteria^12^. Inclusion criteria were as follows: (1) 18 to 65 years old; (2) acute on chronic liver failure caused by hepatitis B virus infection; (3) extreme fatigue and obvious digestive symptoms; (4) progressive increase in jaundice, with serum total bilirubin 10 times higher than the upper limit of normal value or increasing by 1 mg/dL every day; (5) bleeding tendency, with PTA ≤ 40% (or INR ≥ 1.5) and excluding other causes; (6) one or more of the following conditions: hepatorenal syndrome, upper gastrointestinal hemorrhage, severe infection, hepatic encephalopathy; and (7) no previous history of liver transplantation. The exclusion criteria were as follows: (1) liver failure from other causes; (2) liver cancer or other tumors; (3) HIV infection or other immunocompromise states; (4) severe cardiopulmonary diseases or hemodynamic instability; and (5) loss-to-follow-up or incomplete follow-up data.

**Figure 1:**
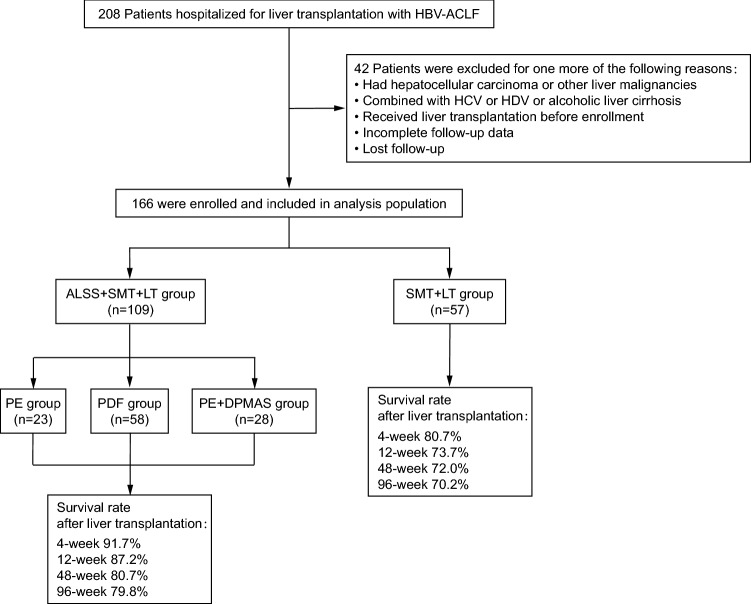
Screening, enrollment and analysis of patients with HBV-ACLF. HBV-ACLF: hepatitis B virus-related acute-on-chronic liver failure; HCV: hepatitis C virus; HDV: hepatitis D virus; ALSS: artificial liver support system; SMT: standard medical therapy; LT: liver transplantation; PE: plasma exchange; PDF: plasma diafiltration; DPMAS: double plasma molecular adsorption system.

According to the inclusion and exclusion criteria, a total of 166 patients were finally enrolled in this retrospective study (Fig. 1). Of the 166 patients, 109 received 322 plasma exchange-centered ALSS treatments plus standard medical therapy (SMT) prior to LT. We divided the patients separated into ALSS + SMT + LT group (observation group), and the remaining 57 patients underwent emergency LT after SMT were allocated to the SMT + LT group (control group). The outcomes were survival rates at 4, 12, 48, and 96 weeks after LT.

### Standard medical therapy (SMT)

All patients were treated with SMT, an integrative medical treatment including management and prevention of complications and treatment of organ failure and other necessary therapies. All patients were given absolute bed rest and nutritional support, hepatocyte protection agents and agents to promote regeneration (glycyrrhizic acid, reduced glutathione, polyene phosphatidylcholine, adenosine methionine, etc.). Patients with positive HBV-DNA were given antiviral therapy with nucleoside analogues (NAs) (including telbivudine, lamivudine, adefovir, entecavir and tenofovir). Patients with infections were treated with appropriate antibiotics. Those with elevated blood ammonia levels or hepatic encephalopathy were treated with ornithine aspartate to reduce blood ammonia levels. Patients with ascites were treated with abdominal paracentesis and diuretics combined with human serum albumin infusion. Some patients were treated with plasma and red blood cell transfusions.

### ALSS treatment

In addition to SMT, patients in the observation group were treated with PE-centered ALSS prior to LT. A double-lumen catheter was used to establish access via the femoral vein or jugular vein. Various methods of ALSS were used by the artificial liver center experts depending on the patients’ conditions and the acquisition of plasma. These methods included PE alone, plasma diafiltration (PDF) and PE with double plasma molecular adsorption (DPMAS). In current study, PE was carried out using membrane-based devices, Cascadeflo EC-40W (Asahi Kasei Medical, Tokyo, Japan). And the blood flow rate was 100–120 ml/min with the plasma exchange rate 18–25 ml/min. PDF was carried out using BLS 816SD hemofilter (Belloco S.r.l., Mirandola, Italy). The blood flow was 120 ml/min and filtration rate was 10–12 ml/min, with filtration volume of 4000–5000 ml. PE was used to deal with coagulopathy and to remove endotoxins. PDF was a combination of PE and diafiltration, and used for hepatic encephalopathy, kidney failure and electrolyte disturbance. PE with DPMAS was used to treat hyperbilirubinemia and remove inflammation mediators (Supplementary Table 1). And plasma substitutes such as albumin to replace plasma and reduce the amount of plasma. The ALSS was usually performed in 24 to 48 h after ACLF diagnosed, and for one or two sessions per week until TB ≤ 5 mg/dl or sustained improvement of coagulation and hyperbilirubinemia, or no ACLF and LT. A total of 322 episodes of ALSS therapy were provided to the observation group, including 77 PE alone, 157 PDF, and 88 PE with DPMAS. The patients were treated with artificial livers eight times at most and one time at least, with a median of 2 and an average of 2.95 times per person.

During treatment, dexamethasone was used to prevent allergy. Heparin and protamine were given for anticoagulation. Finally, patients received special nursing care and ECG monitoring.

### Adverse events of ALSS

All adverse events (AEs) of ALSS treatment were recorded. The overall incidence of AEs was 22.9%(25/109), among which the highest was deep vein thrombosis (44%, 11/25), followed by skin rash or itch (20%, 5/25), nausea or vomiting (16%, 4/25), mouth numbness (12%, 3/25), hypotension (8%, 2/25), bleeding at the puncture site (8%, 2/25), infections (4%, 1/25) and arrhythmia (4%, 1/25). The incidence of 2 or more AEs occurred at the same patient was 16% (4/25).

### LT

All patients were treated with modified piggyback LT. The volume of infusion and blood products were strictly controlled during operation. Acid–base balance, electrolyte and blood coagulation were dynamically monitored during surgery.

### Treatment after LT

After LT, all patients were transferred to the intensive care unit (ICU), where they received comprehensive medical support treatment, then were transferred to general ward after they stabilized. The antiviral regimen was entecavir combined with low-dose hepatitis B immunoglobulin intramuscular injection, and the immunosuppressive regimen was tacrolimus or cyclosporine A combined with mycophenolate mofetil and steroids.

### Data collection

The general data and disease information of the patients before and after treatment were recorded, including age, sex, time of admission and diagnosis, clinical symptoms and changes before and after treatment, Laboratory indicators (e.g., neutrophil, lymphocyte, alanine aminotransferase (ALT), total bilirubin (TB), international normalized ratio (INR), serum creatinine (CR)), complications (including grading of hepatic encephalopathy, gastrointestinal bleeding, hepatorenal syndrome, and secondary infection), LT waiting time, intraoperative blood loss, postoperative time in ICU and total postoperative hospital length of stay, postoperative complications. MELD score, NLR, CLIF-C OF score (Supplementary Table 2), CLIF-C ACLF score and COSSH ACLF score were calculated. The calculation formula of each score was as follows:

- MELD = 9.57 * Ln (Cr mg/dl) + 3.78 * Ln (TBIL mg/dl) + 11.2 * Ln (INR) + 6.43;
- NLR = neutrophil/lymphocyte;
- CLIF-C-ACLF = 10 * [0.33 * CLIF-OFs + 0.04 * Age + 0.63 * Ln(WBC)-2];
- COSSH ACLF = 0.741 * INR + 0.523 * HBV-SOFA + 0.026 * Age + 0.003 * TBIL.

All patients were followed up for 4, 12, 48, and 96 weeks after LT. The end points were as follows: those who died or gave up treatment and were discharged automatically during hospitalization; those lost to follow-up were defined as death cases. The time of death was recorded, as well as the survival time; Patients who did not die after 96 weeks of follow-up (672 days) were defined as surviving patients.

### Statistical analysis

Data were analyzed using SPSS23.0 (SPSS Inc., Chicago, IL, USA) and R v.3.3.2 (https://www.r-project.org)). The results were expressed as mean ± standard deviation (SD), median (interquartile range), frequencies, and percentages. Continuous variables were analyzed using the Student’s t-test or Mann–Whitney U-test. Categorical variables were analyzed using the chi-square test or Fisher test. Independent predictors of short-time mortality were identified using Cox regression models. The survival rates of the two groups were compared using the Kaplan–Meier method. P-value < 0.05 was considered statistically significant.

### Ethical approval

This study was approved by the Clinical Research Ethics Committee of the First Affiliated Hospital, Zhejiang University School of Medicine (No. 2011-13). The precepts of the Declaration of Helsinki were strictly followed for each organ donation and transplant performed in our center. All donors and recipients provided informed written consent. All data were analyzed anonymously.

## Results

### Demographics and clinical characteristics in the two groups

Patients in the observation group were significantly younger than those in the control group (44.75 ± 9.81 vs 48.09 ± 9.40, p < 0.05), with higher ALT/AST, TBil, and TBA levels, meaning patients in the observation group were in poorer condition. There were no significant differences between groups in terms of gender, laboratory indexes, clinical complications, or prognostic scores (Table 1).

**Table 1:** Baseline characteristics of patients at enrolment.

Characteristics	Observation group (n = 109)	Control group (n = 57)	p
Sex (male)	98 (89.9%)	48 (84.2%)	0.412
Age (year)	44.75 ± 9.81	48.09 ± 9.40	0.036
**Laboratory data**			
WBC (10^9^/L)	7.4 [5.3, 10.0]	6.6 [4.4, 8.2]	0.065
NLR	4.8 [2.9, 7.4]	4.8 [2.7, 8.0]	0.942
CRP (mg/L)	11.1[3.1, 19.5]	10.5 [2.6, 17.8]	0.251
PLT (10^9^/L)	113 [79, 147]	84 [45, 127]	0.001
ALB (g/dL)	33 [31, 35]	33 [32, 36]	0.634
ALT (U/L)	485 [173, 854]	219 [124, 542]	0.012
AST (U/L)	287 [134, 623]	179 [104, 295]	0.003
TBA (μmol/L)	210 [169, 257]	182 [136, 240]	0.020
Tbil (μmol/L)	412[345, 495]	366 [266, 469]	0.025
Cr (μmol/L)	63 [53, 73]	68 [55, 86]	0.064
Na^+^ (mmol/L)	138 [135, 140]	137 [134, 141]	0.962
INR	2.3 [2.0, 2.8]	2.3 [2.0, 3.0]	0.981
PTA	0.23 [0.18, 0.30]	0.24 [0.17, 0.29]	0.888
AFP (μg/L)	62.1 [19.9, 178.3]	54.0 [11.8, 177.3]	0.285
**HBV DNA (IU/ML)**			0.128
< 1000	14 (12.8%)	17 (29.8%)	
1000–20,000	14 (12.8%)	6 (10.5%)	
2 × 10^4^–2 × 10^6^	47 (43.1%)	20 (35.1%)	
2 × 10^6^–2 × 10^7^	31 (28.4%)	13 (22.8%)	
NA	3 (2.8%)	1 (1.8%)	
**Scores**			
MELD	24.18 [22.38, 27.34]	25.41 [21.84, 29.94]	0.241
CLIF-C-ACLFs	42.84 ± 6.50	42.35 ± 8.04	0.669
CLIF-C-OFs	10.00 [9.00, 11.00]	10.00 [9.00, 11.00]	0.740
COSSH-ACLF	6.70 [6.18, 7.11]	6.80 [5.94, 7.58]	0.655
**Clinical features**			
Liver cirrhosis	98 (89.9%)	52 (91.2%)	0.998
HE			0.173
0	52 (47.7%)	28 (49.1%)	
1	24 (22.0%)	10 (17.5%)	
2	17 (15.6%)	16 (28.1%)	
3	8 (7.3%)	1 (1.8%)	
4	8 (7.3%)	2 (3.5%)	
Hepatorenal syndrome	10 (9.2%)	8 (14.0%)	0.488
Ascites	63 (57.8%)	36 (63.2%)	0.616
Gastrointestinal bleeding	6 (5.5%)	10 (17.5%)	0.026
Infection	23 (21.1%)	11 (19.3%)	0.944
**ALSS treatment**			
PE	21.1% (23/109)	–	
PDF	53.2% (58/109)	–	
PE + DPMAS	25.7% (28/109)	–	

Data are expressed as mean ± standard deviation (SD), median (interquartile range) or percentages (number of patients). Continuous variables were compared by using Student’s t-test and the Mann–Whitney U test, and the categorical variables were compared using the χ^2^ or Fisher’s exact test between the observation and control groups.

NA: not available; MELD: model for end-stage liver disease; HE: hepatic encephalopathy; CLIF-C: chronic liver failure consortium; ACLF: acute-on-chronic liver failure; CLIF-C-ACLFs: CLIF-C ACLF score; CLIF-C-OFs: CLIF-C organ failure score; COSSH: Chinese Group on the Study of Severe Hepatitis B; PE: plasma exchange; PDF: plasma diafiltration; DPMAS: double plasma molecular adsorption system.

### The efficacy of ALSS

#### Changes in serum parameters and prognostic scores after ALSS

According to the modes of artificial liver support system patients received, 109 patients in the observation group were divided into PE group, PDF group, and PE + DPMAS group. Before ALSS treatment, patients were in poor condition in terms of clinical symptoms and biochemical parameters. After ALSS treatment, conditions in each subgroup improved (Table 2 and Supplementary Fig. 1). Liver and kidney functions, coagulation functions, and electrolyte indexes were significantly better than before the first session of ALSS treatment. Biochemical parameters (included ALT, AST, TBil, INR, Cr, and other indicators), were significantly lower in each subgroup (p < 0.05). MELD and COSSH ACLF scores after treatment were significantly lower as well (p < 0.05). CLIF-C-ACLF scores also showed decreasing trends in each subgroup; however, there was no significant difference (p > 0.05).

**Table 2:** The changes of laboratory parameters and prognostic scores of each subgroup of ALSS.

	PE (n = 23)			PDF (n = 58)			PE + DPMAS (n = 28)		
	Before	After	p	Before	After	p	Before	After	p
WBC (10^9^/l)	9.1 ± 4.5	10.5 ± 3.8	0.10	7.4 ± 4.2	9.2 ± 3.6	< 0.05	8.9 ± 4.2	9.9 ± 4.8	0.09
N (10^9^/l)	7.0 ± 3.7	8.5 ± 3.2	0.07	3.45 ± 3.6	7.5 ± 3.2	< 0.05	6.5 ± 3.9	7.9 ± 4.2	< 0.05
HGB (g/l)	132.0 ± 29.4	119.6 ± 21.8	< 0.05	135.8 ± 21.2	114.6 ± 21.8	< 0.05	134.3 ± 20.7	114.1 ± 17.7	< 0.05
PLT (10^9^/l)	106.4 ± 38.8	88.4 ± 39.3	< 0.05	118.9 ± 52.3	87.3 ± 46.1	< 0.05	132.6 ± 59.3	99.1 ± 60.4	< 0.05
ALB (g/dl)	33.0 ± 4.5	33.9 ± 3.5	0.32	32.9 ± 4.0	33.7 ± 3.6	0.29	33.1 ± 5.2	33.2 ± 3.5	0.93
ALT (U/L)	443.7 ± 376.4	203.9 ± 181.8	< 0.05	833.5 ± 781.0	174.6 ± 195.3	< 0.05	576.3 ± 765.3	139.4 ± 208.9	< 0.05
AST (U/L)	281.7 ± 266.1	187.3 ± 212.0	< 0.05	660.5 ± 653.4	131.0 ± 113.8	< 0.05	419.6 ± 494.9	96.9 ± 52.6	< 0.05
TBA (μmol/l)	190.5 ± 64.8	161.0 ± 87.9	0.07	219.1 ± 67.9	188.3 ± 66.9	< 0.05	232.9 ± 69.2	181.9 ± 64	< 0.05
TBil (μmol/l)	447.9 ± 109.2	335.6 ± 106.3	< 0.05	420.0 ± 122.6	317.7 ± 112.0	< 0.05	407.5 ± 153.6	301.9 ± 127.8	< 0.05
Cr (μmol/l)	77.6 ± 50.2	74.4 ± 51.3	0.53	62.5 ± 20.5	52.3 ± 20.7	< 0.05	73.6 ± 33.2	64.7 ± 28.7	< 0.05
Na^+^ (mmol/l)	137.4 ± 4.8	136.5 ± 3.8	0.41	137.8 ± 4.1	136.1 ± 4.6	< 0.05	137.4 ± 2.9	136.3 ± 2.8	0.10
INR	2.6 ± 0.6	2.1 ± 0.8	< 0.05	2.5 ± 0.6	1.9 ± 0.6	< 0.05	2.3 ± 0.8	2.2 ± 0.7	0.19
PTA	0.23 ± 0.08	0.32 ± 0.16	< 0.05	0.25 ± 0.10	0.34 ± 0.13	< 0.05	0.27 ± 0.09	0.30 ± 0.12	0.12
NLR	6.56 ± 3.75	4.81 ± 1.87	< 0.05	5.58 ± 3.43	5.29 ± 2.62	0.58	5.0 ± 3.3	4.7 ± 2.6	0.07
MELD	26.7 ± 4.9	22.6 ± 6.7	< 0.05	24.4 ± 3.8	19.7 ± 4.7	< 0.05	24.6 ± 6.1	21.4 ± 6.8	< 0.05
CLIF-C-ACLFs	45.9 ± 7.9	44.8 ± 7.8	0.27	42.3 ± 5.7	41.2 ± 6.8	0.13	41.4 ± 6.0	41.0 ± 7.1	0.63
COSSH-ACLFs	7.1 ± 0.9	6.5 ± 1.1	< 0.05	6.7 ± 0.6	6.0 ± 0.7	< 0.05	6.6 ± 1.0	6.1 ± 1.0	< 0.05

Data are expressed as mean ± standard deviation(SD) and were compared using the Student’s t-test.

ALSS: artificial liver support system; PE: plasma exchange; PDF: plasma diafiltration; DPMAS: double plasma molecular adsorption system; WBC: White blood cell; N: neutrophil; HGB: hemoglobin; PLT: platelet; ALB: albumin; ALT: alanine aminotransferase; AST: aspartate aminotransferase; TBA: total bile acid; TBil: total bilirubin; Cr: creatinine; Na^+^: blood sodium; INR: international normalized ratio; PTA: prothrombin activity; MELD: model for end-stage liver disease; CLIF-C: chronic liver failure consortium; ACLF: acute-on-chronic liver failure; CLIF-C-ACLFs: CLIF-C ACLF score; CLIF-C-OFs: CLIF-C organ failure score; COSSH: Chinese Group on the Study of Severe Hepatitis B.

#### Impacts of ALSS treatment on operation

Compared with the control group (Table 3), patients in the observation group had significantly longer waiting times for liver donation (21.24 ± 21.1 d vs 5.23 ± 5.73 d, p < 0.05). Blood loss during surgery and ICU times after surgery were both significantly lower (1306.42 ± 969.56 ml vs 1843.86 ± 1311.08 ml, p < 0.05; 9.79 ± 5.68 d vs. 10.73 ± 4.62 d, p < 0.05). There was no significant difference in the total postoperative hospital staying time, mean anhepatic time and mean operation time between two groups (p > 0.05). Patients in the control group tended to have more severe complications (p < 0.05).

**Table 3:** Impacts of artificial liver support system treatment on operation.

	Observation group (n = 109)	Control group (n = 57)	p
Waiting time for liver donor (days)	14 [7, 29]	3 [2, 6]	< 0.001
Median time to transplant after last session of ALSS	4 [2, 10]	3 [2, 6]	> 0.05
Intraoperative blood loss (ml)	1000 [600, 1500]	1500 [1000, 2200]	< 0.001
Anhepatic time (min)	61.2 ± 16.75	64.91 ± 15.36	> 0.05
Operation time (min)	368.25 ± 69.12	359.18 ± 72.37	> 0.05
ICU staying time (days)	8 [7, 12]	10 [8, 13]	< 0.05
Postoperative hospital staying time (days)	24 [20, 30]	25 [21, 30]	0.917
**Postoperative severe complications**			
0	69 (63.3%)	35 (61.4%)	0.070
1	24 (22.0%)	6 (10.5%)	< 0.05
2	12 (11.0%)	10 (17.5%)	< 0.05
3	4 (3.7%)	4 (7.0%)	< 0.05
≥ 4	0 (0.0%)	2 (3.5%)	< 0.05

Data are expressed as mean ± standard deviation (SD), median (interquartile range) or percentages (number of patients). Postoperative severe complications include: liver failure, renal failure, circulatory failure, septic shock, etc.

ALSS: artificial liver support system; ICU: Intensive care unit.

#### Impacts of ALSS treatment on patients’ short-time survival after LT

**Figure 2:**
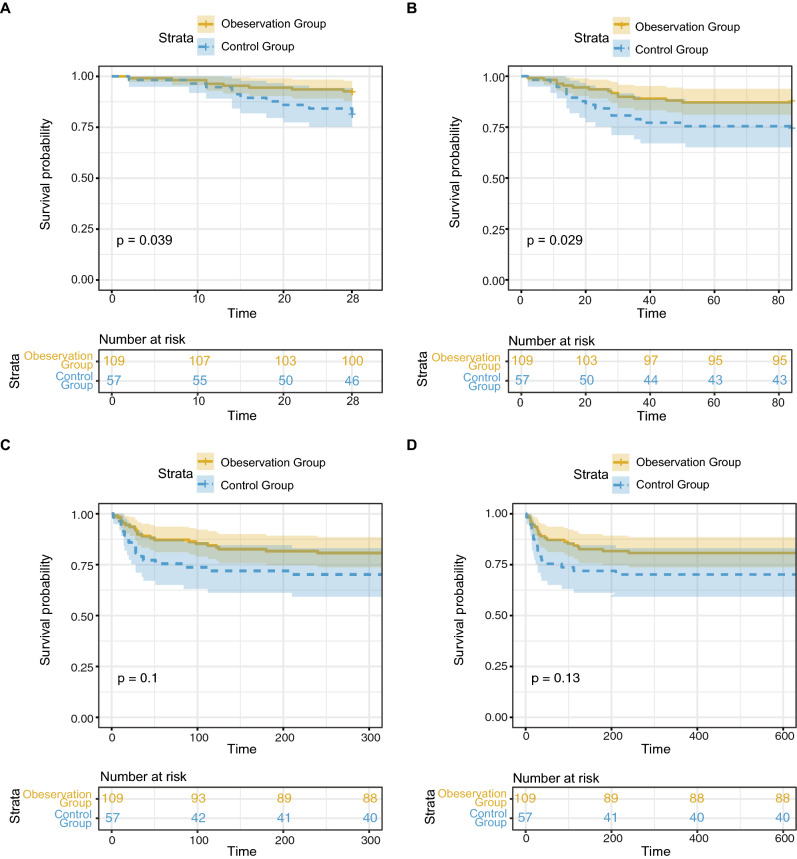
Kaplan–Meier curves for short-term survival in the observation group and control groups. (**A**) The 4-week survival curve after LT; (**B**) The 12-week survival curve after LT; (**C**) The 48-week survival curve after LT; (**D**) The 96-week survival curve after LT.

Overall 4-week, 12-week, 48-week, and 96-week survival rates after LT were 88.0%, 82.5%, 77.1%, and 76.5%, respectively (Fig. 2). The 4- and 12-week survival rates in the observation group were significantly higher than those of the control group (91.7% vs. 80.7%, 87.2% vs. 73.7%, P < 0.05). The 48- and 96-week survival rates in the observation group were also higher than those of the control group; however, there was no significant difference (80.7% vs. 72.0%, 79.8% vs. 70.2%, P > 0.05). Furthermore, there was no significant difference between subgroups of ALSS in short-term as well as overall survival rates (p > 0.05) (Supplementary Fig. 2).

#### ALSS treatment pre-LT was an independent predictor of short-time survival

Taking the survival status at 4 weeks after LT as the end point, all 166 patients were divided into a survival group (n = 146) and a death group (n = 20), and the prognostic factors associated with the groups were analyzed using univariate and multivariate COX regression analyses (supplementary Table 3). We found that Preoperative ALSS treatment was an independent protective factor for reducing 4-week mortality of HBV-ACLF patients after LT (HR: 0.314, 95% CI 0.128–0.773, p < 0.05). Preoperative NLR value and intraoperative blood loss were independent risk factors for increasing the 4-week mortality of HBV-ACLF patients after LT (HR: 1.266, 95% CI 1.119–1.431, and HR: 1.418, 95% CI 1.128–1.784, respectively, P < 0.05).

#### Predictive value of each prognostic models for short-term prognosis (4-week survival) after LT

**Figure 3:**
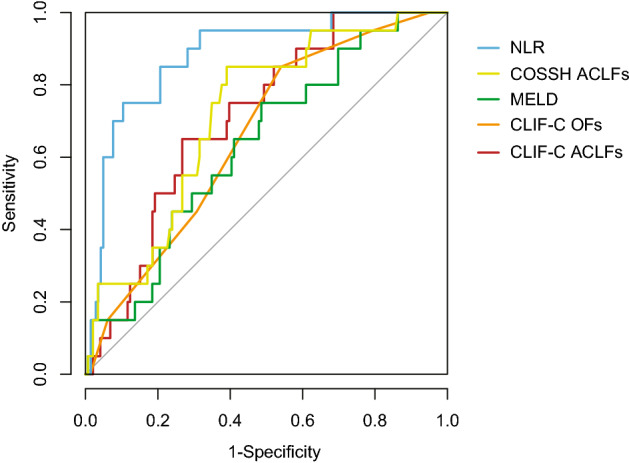
Receiving operating characteristics (ROC) curves for the abilities of prognostic models to predict the 4-week prognosis of patients with HBV-ACLF after liver transplantation. ACLF: acute-on-chronic liver failure; MELD: Model for end-stage liver disease; CLIF-C: chronic liver failure consortium; CLIF-C-ACLFs: CLIF-C ACLF score; CLIF-C-OFs: CLIF-C organ failure score; COSSH: Chinese Group on the Study of Severe Hepatitis B. COSSH ACLFs: COSSH ACLF score; NLR: neutrophil–lymphocyte rate.

To compare the predictive values of NLR, COSSH ACLF, MELD, CLIF-C ACLF, and CLIF-C OFs for estimating 4-week prognosis after LT of patients with HBV-ACLF, we analyzed the ROC curves of these five parameters (Fig. 3). The area under curve (AUC) of NLR (AUROC: 0.882) was significantly higher than that of COSSH ACLF (AUROC: 0.72), MELD (AUROC: 0.64), CLIF-C ACLF (AUROC: 0.71), or CLIF-C OFs (AUROC: 0.66). We calculated the cut-off value of each ROC curve of the prognostic models and then split the patients into two groups according to the cut-off value. Patients with NLR ≥ 8.5 or COSSH ACLF ≥ 6.79 or MELD ≥ 28.0 or CLIF-C ACLF ≥ 45.87 or CLIF-C OFs ≥ 10.0 had significantly higher mortality (p < 0.05) (Supplementary Fig. 3).

## Discussion

HBV-ACLF is a common clinical end-stage liver disease. It is characterized by rapid progression and is accompanied by several complications and multiple organs failure with high short-term mortality^1,13^. At present, there is no effective treatment for HBV-ACLF. Nevertheless, thanks to the rapid development of LT in recent decades, short-term mortality of patients with HBV-ACLF after LT has been significantly reduced. In some large LT centers, the 1-year survival rate after LT was more than 70%^7^, and in the best centers, the proportion has been 80–90%^14^. However, because of the critical shortage of donor livers and the high cost of LT, most patients do not undergo LT in a timely fashion. Conditions deteriorate in these patients and severe complications result. Many die while waiting for a donor liver. Halting or retarding the progression of CHB to ACLF is the most effective way to reduce the morbidity and mortality associated with HBV-ACLF. For this reason, temporary liver support system therapy is critical for patients with HBV-ACLF. ALSS is an extracorporeal liver support system that has become increasingly popular over more than 20 years. As an important part of ALSS, PE has been most commonly used in liver failure to remove endotoxins and large molecules of protein-bound toxins and ameliorate the severe coagulopathy. For medium and small molecules, such as ammonia, aromatic amino acids and creatinine, PDF (combination of PE and diafiltration) is often used. DPMAS serves to remove medium and large molecules, including bilirubin, albumin-bound toxins and inflammation mediators^8,9^. Patient conditions can be improved rapidly in a short period of time using ALSS, thereby gaining to bridge to LT^15–18^. Non-bioartificial liver (NBAL) combined with integrative medical treatment effectively improved the condition of HBV-ACLF patients and reduced short-term mortality^8,16,19^. After NBAL treatment, MELD scores of HBV-ACLF patients can be significantly reduced, thereby prolonging the waiting time for donors and improving survival rate of patients waiting for LT^20,21^. Although some studies suggest that the support of ALSS to the liver is only temporary, and the index of liver failure will still rebound after the loss of ALSS support, approaching or even exceeding the level before ALSS treatment^22^, it remains an important tool to stabilize the disease, to recover liver function and to regenerate hepatocytes in the short term, allowing patients to transit smoothly to LT.

In the observation group, all patients were treated with PE-centered ALSS. Our results support those of previous studies^23,24^. Before ALSS treatment, 109 patients were in poor conditions and complicated with hepatic encephalopathy, or hepatorenal syndrome, infection, and others. After ALSS treatment, liver and kidney function, inflammatory factors, and coagulation functions significantly improved; MELD, COSSH ACLF, and CLIF-C ACLF scores decreased significantly. Compared with the control group, ALSS not only improved conditions before surgery, but also had significant positive effects during and after surgery, including on blood loss during surgery and ICU days. In addition to observing significant improvements in biochemical indicators and clinical symptoms, we also found that ALSS therapy improved short-term survival rates after LT of patients with HBV-ACLF. The rate of severe complications after LT in the observation group were also significantly lower than those in the control group. This suggests that ALSS therapy is as an important measure before LT to not only remove the endotoxins, cytokines, inflammation mediators, but also improve the intraoperative tolerance. For patients who are waiting for LT, especially those who cannot obtain liver donors in a short period of time, timely ALSS treatment should be considered.

In view of the fact that the pathogenesis of HBV-ACLF is related to systemic inflammatory response syndrome^2,25,26^, the NLR reflects the degree of inflammation. Previous studies have found that NLR was an independent prognostic factor for HBV-ACLF patients treated with ALSS; NLR > 6 indicated poor prognosis and required emergency LT^27^. In our study, NLR also predicted short-term prognosis of patients with HBV-ACLF. When preoperative NLR ≥ 8.5, the short-term prognosis after LT was poor (sensitivity 75%, specificity 89.7%). For these patients, it is necessary to comprehensively analyze the possibility of postoperative benefits before surgery.

Taken together the results of this study suggest that preoperative ALSS combined with LT therapy effectively improves short-term survival in HBV-ACLF patients after LT. ALSS treatment had a significant effect on hepatic and renal function, electrolytes, coagulation, and inflammatory indexes. ALSS safely and effectively prolonged waiting times for liver donation, intraoperative blood loss, and postoperative recovery.

Although prospective large-sample from multicenter randomized controlled trials are still needed to be conducted to further verify the beneficial effects of ALSS on post-LT, this study undoubtedly provides a reference and support for the future research.

## Conclusion

ALSS treatment combined with LT in patients with HBV-ACLF improves the short-term survival rate. ALSS treatment pre-LT is an independent protective factor affecting 4-week survival rate after LT. NLR, COSSH-ACLF, MELD, CLIF-C-ACLF, and CLIF-C OF scores before LT affected the short-term prognosis of HBV-ACLF patients. Of these, preoperative NLR value is the most valuable for predicting short-term survival after LT. This needs to be further verified in multicenter large sample prospective cohort studies.

## Supplementary Information

Supplementary Information.

## Abbreviations

ALT: Alanine aminotransferase
AST: Aspartate aminotransferase
TBil: Total bilirubin
Cr: Creatinine
INR: International normalized ratio
ACLF: Acute-on-chronic liver failure
ALSS: Artificial liver support system
NLR: Neutrophil–lymphocyte ratio
MELD: Model for end-stage liver disease
CLIF-C: Chronic liver failure consortium
COSSH: Chinese Group on the Study of Severe Hepatitis B
