# Supplementary material for: A non-bioartificial liver support system combined with transplantation in HBV-related acute-on-chronic liver failure
Source: Sci Rep. 2021 Feb 3;11:2975. doi: 10.1038/s41598-021-82719-x (PMC7859234; doi:10.1038/s41598-021-82719-x)
Supplement: Supplementary file 1 — Supplementary Information. [file 41598_2021_82719_MOESM1_ESM.pdf]

A non-bioartificial liver support system combined with transplantation in HBV-related acute-on-chronic liver failure

Peng Li[1], Xi Liang[2], Shan Xu[1], Ye Xiong[1]， Jianrong Huang[1]

Contents

- Supplementary Figure 1: The clinical efficacy of each subgroup of artificial liver support system (ALSS).
- Supplementary Figure 2: Kaplan-Meier curves for the overall survival of patients in subgroups of ALSS after liver transplantation.
- Supplementary Figure 3: Kaplan-Meier curves for the 4-week survival after liver transplantation.
- Supplementary Table 1: Description of different modes of ALSS.
- Supplementary Table 2: CLIF-C OF score.
- Supplementary Table 3: Univariate and multivariate COX regression analysis investigating independent factors on 4-week prognosis in HBV-ACLF patients after LT.

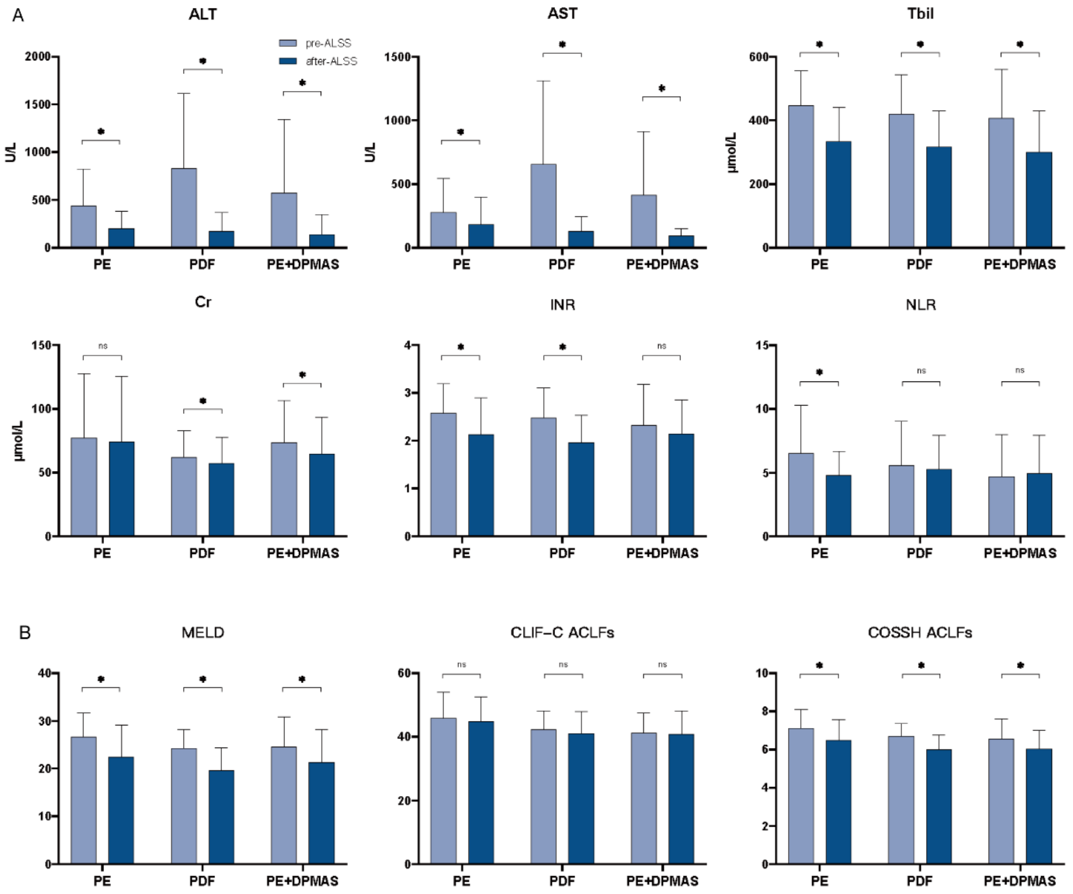

Supplementary Fig 1. The clinical efficacy of each subgroup of artificial liver support system (ALSS). (A)changes in laboratory parameters after ALSS; (B)changes in prognostic scores after ALSS. PE: plasma exchange; PDF: plasma diafiltration; DPMAS: double plasma molecular adsorption system; NLR: neutrophil-lymphocyte ratio; MELD: Model for end-stage liver disease; CLIF-C: chronic liver failure consortium; ACLF: acute-on-chronic liver failure; CLIF-C-ACLFs: CLIF-C ACLF score; CLIF-C-OFs: CLIF-C organ failure score; COSSH: Chinese Group on the Study of Severe Hepatitis B.

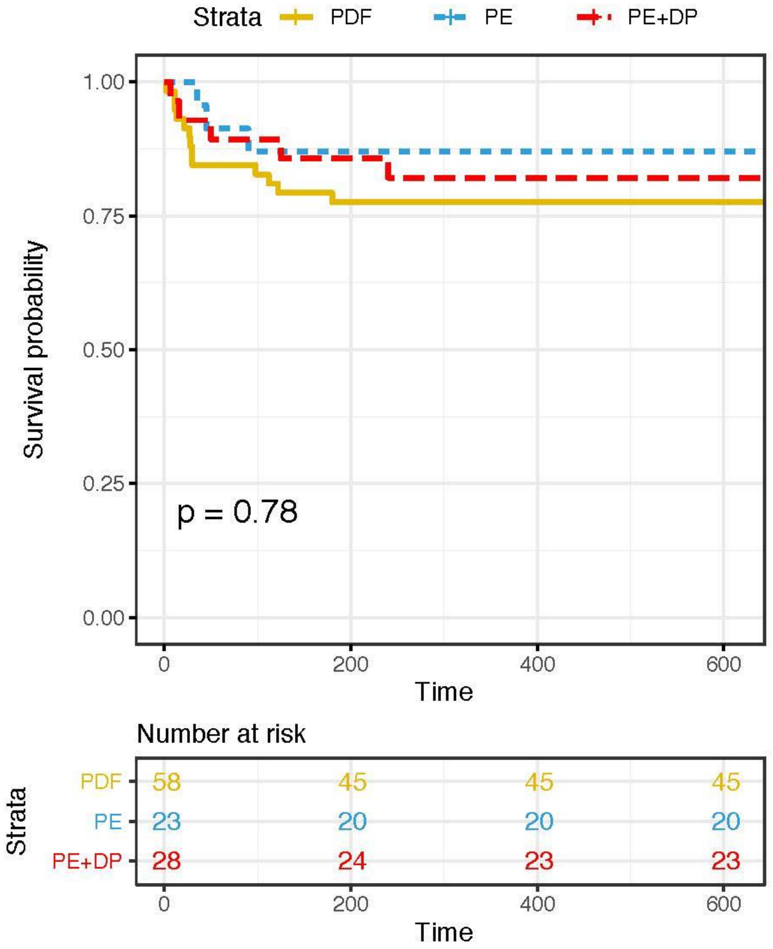

Supplementary Fig 2. Kaplan-Meier curves for the overall survival of patients in subgroups of ALSS after liver transplantation. PE: plasma exchange; PDF: plasma diafiltration; DPMAS; double plasma molecular adsorption system.

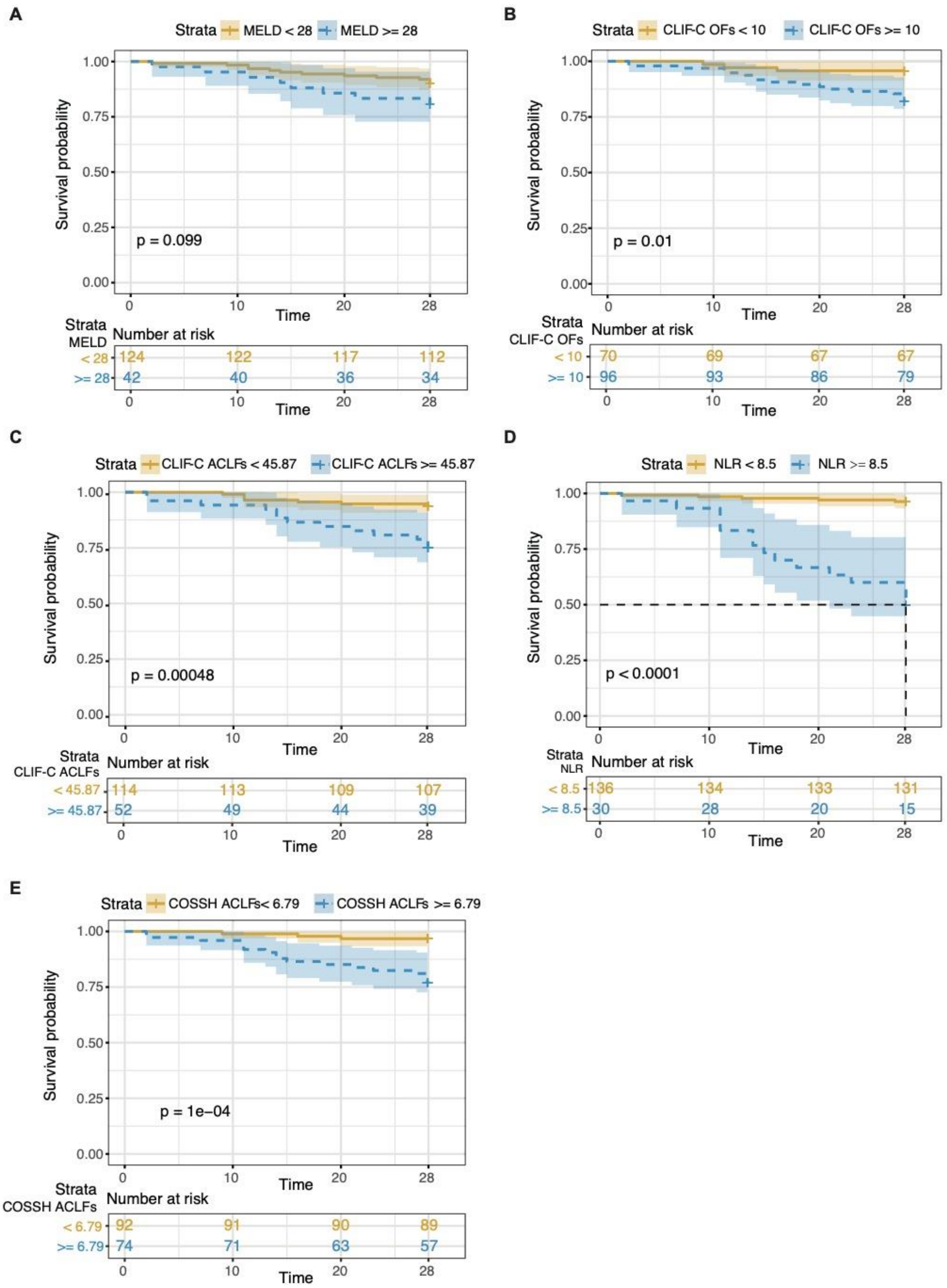

Supplementary Fig 3. Kaplan-Meier curves for the 4-week survival after liver transplantation. (A) MELD >=28 or MELD <28; (B) CLIF-C OFs >=10 or CLIF-C OFs <10; (C) CLIF-C ACLFs >= 45.87 or CLIF-C ACLFs <45.87; (D) NLR >=8.5 or NLR <8.5; (E) COSSH-ACLFs >=6.79 or COSSH-ACLFs <6.79. ACLF: acute-on-chronic liver failure; MELD: Model for end-stage liver disease; CLIF-C: chronic liver failure consortium; CLIF-C-ACLFs: CLIF-C ACLF score; CLIF-C-OFs: CLIF-C organ failure score; COSSH: Chinese Group on the Study of Severe Hepatitis B. COSSH ACLFs: COSSH ACLF score; NLR: neutrophil-lymphocyte ratio.

Supplementary Table 1. Description of different modes of ALSS

Parameters	Modes		
	PE	PDF	DPMAS
Blood flow rate (ml/min)	100-120	120	120
Rate (ml/min)	Plasma exchange rate:18-25	Exchange rate:3-4<br>Dialysis rate:15-16<br>Filtration rate:10-12	Plasma separation: 25-30
Blood volume (ml)	Exchange:2000-2500	Exchange:2000<br>Dialysis:8000<br>Filtration:4500-5000	Plasma absorption: 5000-6000
Process	Remove metabolic toxins(small and medium molecules), protein-bound toxins(large molecules), supplement essential substances(such as albumin and coagulation factors), improve internal environment disturbance	Remove water-soluble substances: ammonia, aromatic amino acids and creatinine(medium and small molecules), maintain hemodynamic and electronic balance stable	Remove bilirubin, albumin-bound toxins and inflammation mediators (medium and large molecules)
Indications	Coagulopathy, hyperbilirubinemia	Hepatic encephalopathy, kidney failure and electrolyte disturbance	Hyperbilirubinemia

ALSS: artificial liver support system; PE: plasma exchange; PDF: plasma diafiltration; DPMAS; double plasma molecular adsorption system.

Supplementary Table 2. CLIF-C OF score

Organ/score	1	2	3
Liver (TBil)	<6mg/dl	6-12mg/dl	≥12mg/dl
Kidney (creatinine)	<2mg/dl	2-3.5mg/dl	≥3.5mg/dl
HE grade	0	1-2	3-4
Coagulation (INR)	<2.0	2.0-2.5	≥2.5
Circulation (MAP)	≥70mmHg	<70mmHg	Vasopressors
Respiratory			
PaO_2/FiO_2	>300	200-300	≤200
SpO_2/FiO_2	>357	214-357	≤214

TBil: total bilirubin; HE: hepatic encephalopathy; INR: international normalized ratio; MAP: mean arterial pressure; PaO_2:partial pressure of oxygen; FiO_2: fraction of inspired oxygen; SpO_2: peripheral capillary oxygen saturation.

Supplementary Table 3. Univariate and multivariate COX regression analysis investigating independent factors on 4-week prognosis in HBV-ACLF patients after LT

Variable	Comparison	Univariate analysis		Multivariate analysis	
		HR (95% CI)	P	HR (95% CI)	P
Age (years)	Increase per year	1.06 (1.01-1.11)	0.017	1.934 (0.696-5.377)	0.206
Sex	Male vs. female	0.76 (0.22-2.60)	0.45		
ALT	Increase per U/L	1.00 (1.00-1.00)	0.623		
TBil	Increase per µmol/L	1.00 (1.00-1.00)	0.362		
AST	Increase per U/L	1.00 (1.00-1.00)	0.688		
ALB	Increase per g/L	0.90 (0.82-1.00)	0.044		
Cr	Increase per µmol/L	1.00 (1.00-1.01)	0.462		
INR		1.26 (0.91-1.75)	0.168	2.60 (0.92-7.344)	0.071
WBC	Increase per log /L	1.02 (0.91-1.13)	0.775		
CRP	Increase per mg/L	1.15 (1.01-1.26)	0.051		
HE	Increase per grade	1.48 (1.08-2.01)	0.013	1.366 (0.944-1.976)	0.098
HRS	Yes or no	1.33 (0.93-4.03)	0.928		
Ascites	Yes or no	4.12 (1.21-14.07)	0.024		
ICU stay time	Increase per day	1.01 (0.95-1.07)	0.698		
Intraoperative blood loss	Increase per 500 ml	1.21 (1.01-1.53)	0.023	1.418 (1.128-1.784)	0.003
ALSS pre-LT	Yes or no	0.41 (0.17-0.98)	0.046	0.314 (0.128-0.773)	0.012
NLR	Increase per ratio	1.33 (1.21-1.47)	<0.001	1.266 (1.119-1.431)	<0.001
MELDs		1.07 (1.01- 1.15)	0.032		
COSSH ACLFs		1.57 (1.17- 2.12)	0.003		
CLIF-C OFs		1.49 (1.05- 2.11)	0.024		
CLIF-C ACLFs		1.09 (1.03- 1.16)	0.005		

ALT: alanine aminotransferase; AST: aspartate aminotransferase; ALB: albumin; TBil: total bilirubin; Cr: creatinine; INR: international normalized ratio; WBC: White blood cell; CRP: C-reactive protein; HE: hepatic encephalopathy; HRS: hepatorenal syndrome; ACLF: acute-on-chronic liver failure; ALSS: artificial liver support system; LT: liver

transplantation; NLR: neutrophil-lymphocyte ratio; MELD: Model for end-stage liver disease; CLIF-C: chronic liver failure consortium; CLIF-C ACLFs: CLIF-C ACLF score; CLIF-C OFs: CLIF-C organ failure score; COSSH: Chinese Group on the Study of Severe Hepatitis B; HR: hazard ratio; 95% CI: 95% confidence interval.
